# **Factors influencing failure to undergo interval cholecystectomy after percutaneous cholecystostomy among patients with acute cholecystitis**: **a retrospective study**

**DOI:** 10.1186/s12876-021-01989-x

**Published:** 2021-10-29

**Authors:** Peng Yao, Zhihui Chang, Zhaoyu Liu

**Affiliations:** Department of Radiology, Shengjing Hospital of China Medical University, No. 36, Sanhao Street, Heping District, Shenyang, 110004 China

**Keywords:** Acute cholecystitis, Percutaneous cholecystostomy, Cholecystectomy

## Abstract

**Background:**

Percutaneous cholecystostomy (PC) with interval cholecystectomy is an effective treatment modality in high-risk patients with acute cholecystitis. However, some patients still fail to undergo interval cholecystectomy after PC, with the reasons rarely reported. Hence, this study aimed to explore the factors that prevent a patient from undergoing interval cholecystectomy.

**Methods:**

Data from patients with acute cholecystitis who had undergone PC from January 1, 2017 to December 31, 2019 in our hospital were retrospectively collected. The follow-up endpoint was the patient undergoing cholecystectomy. Patients who failed to undergo cholecystectomy were followed up every three months until death. Univariate and multivariate analyses were performed to analyze the factors influencing failure to undergo interval cholecystectomy. A nomogram was used to predict the numerical probability of non-interval cholecystectomy.

**Results:**

Overall, 205 participants were identified, and 67 (32.7%) did not undergo cholecystectomy during the follow-up period. Multivariate analysis revealed that having a Tokyo Guidelines 2018 (TG18) grade III status (odds ratio [OR]: 3.83; 95% confidence interval [CI]: 1.27–11.49; *p* = 0.017), acalculous cholecystitis (OR: 4.55; 95% CI: 1.59–12.50; *p* = 0.005), an albumin level < 28 g/L (OR: 4.15; 95% CI: 1.09–15.81; *p* = 0.037), and a history of malignancy (OR: 4.65; 95% CI: 1.62–13.37; *p* = 0.004) were independent risk factors for a patient’s failure to undergo interval cholecystectomy. Among them, the presence of a history of malignancy exhibited the highest influence in the nomogram for predicting non-interval cholecystectomy.

**Conclusions:**

Having a TG18 grade III status, acalculous cholecystitis, severe hypoproteinemia, and a history of malignancy influence the failure to undergo cholecystectomy after PC in patients with acute cholecystitis.

## Background

Acute cholecystitis (AC) is a common surgical emergency. Its pathophysiology is mainly derived from the obstruction of the cystic duct of the gallbladder or dyskinesia of its wall, which can lead to increased intraluminal pressure, wall edema, wall ischemic necrosis, or perforation, thereby increasing the probability of bacterial reproduction in the cavity [1]. Although laparoscopic cholecystectomy (LC) is the gold standard for the treatment of AC, LC may be accompanied by high morbidity and mortality in cases of acute inflammation or in high-risk patients, such as the elderly, those with multiple comorbidities, or the critically ill [2–5].

Percutaneous cholecystostomy (PC) is an effective method for treating moderate or severe AC in high-risk patients. Its purpose is to adequately drain the gallbladder to resolve the acute course and prevent local and systemic complications. Related research indicates that PC followed by delayed cholecystectomy has better clinical outcomes than emergency LC [6–8]. Therefore, PC is being increasingly utilized as a bridging therapy before definitive therapy [9].

Some guidelines suggest that patients should undergo interval cholecystectomy to prevent the relapse of AC following the resolution of AC symptoms through PC [10]. However, some patients still fail to undergo interval cholecystectomy because of various reasons. To the best of our knowledge, there are few reports regarding the factors that prevent patients from undergoing interval cholecystectomy.

Therefore, in this study, we retrospectively analyzed the medical records of patients with cholecystitis who had undergone PC in our tertiary teaching hospital, followed up on the subsequent definitive management of the patients initially treated with PC, and explored the factors influencing their failure to undergo interval cholecystectomy.

## Methods

### Study participants

This study was a retrospective review of the data of all patients with AC treated via PC at the Shengjing Hospital of China Medical University, between January 1, 2017 and December 31, 2019. We obtained the demographic and clinical information of patients with cholecystitis from the hospital information system. Consecutive data were collected through a nonselective process.

Patients diagnosed with AC according to the Tokyo Guidelines 2018 (TG18) and who had undergone PC after multidisciplinary evaluation were included [11], and those who had concomitant acute pancreatitis or were lost to follow-up were excluded from the analysis. This study was approved by the ethics committee of our hospital (approval number: 2020PS704K). The requirement of obtaining informed consent from the patients was waived owing to the retrospective nature of the study.

**Fig. 1:**
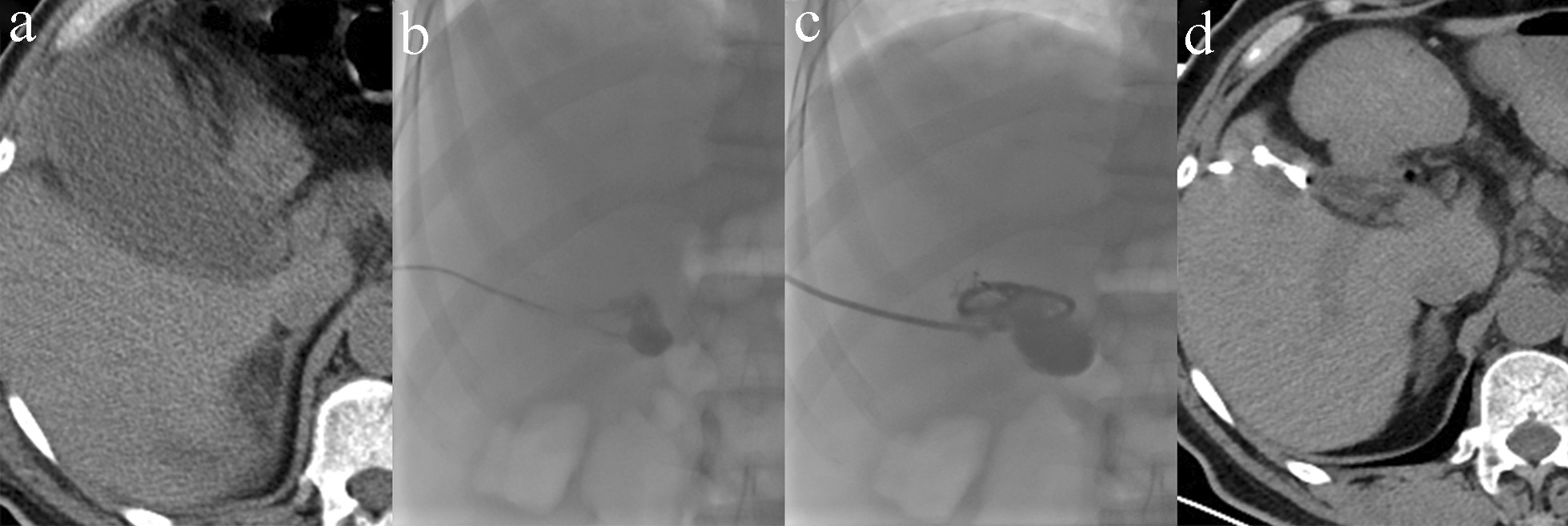
The procedure of percutaneous cholecystostomy. **a** A 59-year-old man was diagnosed with acute acalculous cholecystitis on the 7th day after colon cancer surgery. Computed tomography (CT) showed gallbladder dilatation and gallbladder wall thickening with peripheral exudation. **b** The patient could not tolerate cholecystectomy; therefore, percutaneous cholecystostomy was performed to relieve the patient's symptoms. Ultrasound guided percutaneous puncture of the gallbladder was successfully confirmed by contrast injection. **c** An 8F pigtail drainage catheter was introduced into the gallbladder under fluoroscopic guidance. **d** Three months later, CT showed that the gallbladder had retracted, and the surrounding exudation was absorbed. However, the patient was still unable to tolerate cholecystectomy and had a long-term indwelling PC tube

All PC procedures were performed by interventional radiologists under the guidance of ultrasound combined with fluoroscopy, with 100% technical success. A 5F puncture needle (Cook, USA) was introduced into the gallbladder using the Seldinger technique with real-time sonographic visualization (TERASON2000, Terason, USA). Thereafter, an 8F pigtail multiuse drainage catheter (Bard, USA) was introduced into the cystic cavity under fluoroscopic guidance (Fig. 1).

### Variables and definitions

Based on the literature and our clinical experience, variables were defined as follows. PC-related complications included clogged and dislodged tubes. All-cause death referred to the death of the patient during the follow-up period. History of malignancy referred to the diagnosis of various malignant tumors before performing PC, including previous history of malignancy and current malignancy. Kidney disease referred to all diseases that cause structural damage or dysfunction of the kidney, which mainly include glomerulonephritis, nephrotic syndrome, secondary nephropathy, chronic kidney disease, and chronic renal failure (CKD stage ≥ 3 or glomerular filtration rate < 60 ml/(min·1.73 m^2^)). Patients with any of the following diseases were defined as having cardiovascular and cerebrovascular diseases (CCVD): heart failure, coronary atherosclerotic heart disease, myocardial infarction, arrhythmia, cerebral ischemia, cerebral hemorrhage, and stroke [12].

### Follow-up

All patients who had undergone PC therapy were followed up. If the patient had undergone cholecystectomy, the follow-up was terminated. For patients who did not undergo cholecystectomy, we recommended follow-ups at our interventional radiology clinic every three months. If a patient was unable to visit the clinic, follow-up by phone was performed until the patient’s death.

### Statistical analyses

Continuous variables that satisfied the normal distribution were expressed as mean ± standard deviation. Categorical variables were expressed as percentages and frequencies. Discrete variables were presented as medians and interquartile ranges (IQRs). Continuous or discrete variables were compared using the Mann–Whitney U test or the Kruskal–Wallis test. Categorical data were analyzed using the chi-square test or Fisher’s exact test, as appropriate. Subjects were divided into two groups according to whether they had undergone interval cholecystectomy. To compare the differences between the two groups, we set non-interval cholecystectomy as a column stratification variable and the remaining variables as analysis variables to obtain a description of the study population between the two groups. Multivariate logistic regression models were used to identify independent risk factors associated with the patients’ failure to undergo cholecystectomy during follow-up, and a nomogram was constructed on this basis. We adjusted for potential confounding factors such as age, sex, and candidate variables with *p* < 0.05 in the univariate analysis. The results are presented as odds ratios (ORs) and 95% confidence intervals (CIs). A *p*-value < 0.05 was considered statistically significant. All analyses were performed using Empower (R) (www.empowerstats.com, X&Y solutions, Inc.) and R (http://www.R-project.org; version 3.4.3).

## Results

### Baseline characteristics of the patients

**Fig. 2:**
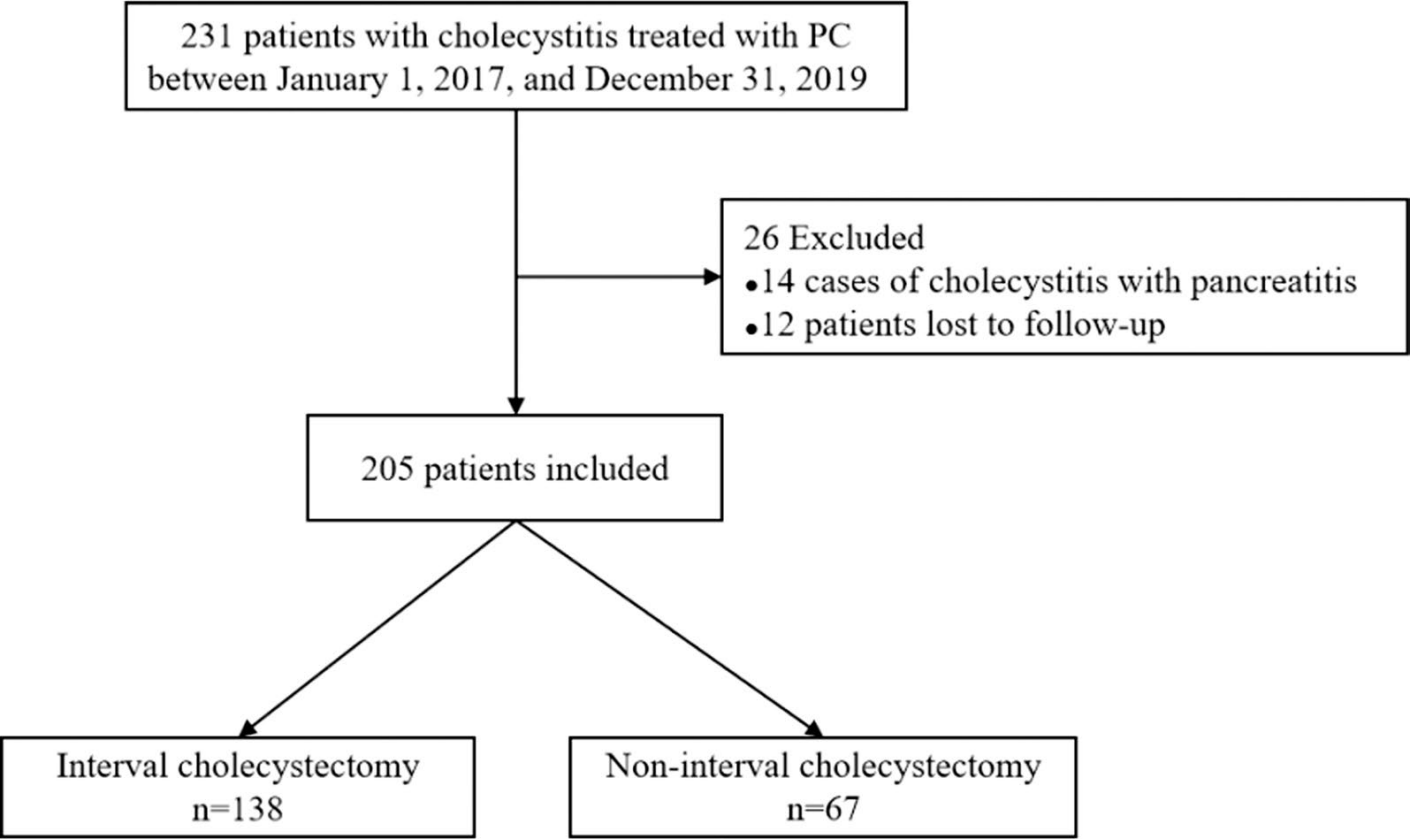
Flowchart of the participant selection process

Overall, 205 patients were selected in this study. The patient selection flowchart is presented in Fig. 2. The average age of the patients was 67.3 ± 14.7 years, and 53.7% of them were male. The patients were followed up until February 7, 2021, and 138 of the 205 patients underwent interval cholecystectomy. The average time from PC to cholecystectomy was 3.3 months. Overall, 67 patients did not undergo interval cholecystectomy, including 21 who had long-term indwelling PC tubes and 46 who had their drainage tubes removed upon meeting the requirements in the clamping tests [13, 14]. The participants’ demographic and clinical characteristics with respect to whether they underwent interval cholecystectomy are shown in Table 1. The average age of the patients who did not undergo interval cholecystectomy was higher than that of those who underwent cholecystectomy (73.40 ± 12.97 vs. 64.33 ± 14.61 years; *p* < 0.001). Significant differences in terms of the TG18 grade, albumin level, presence of gallstones, presence of CCVD, history of malignancy, and number of all-cause deaths were observed between the two groups.

**Table 1:** Demographic and clinical characteristics of the patients included in the study

	Interval cholecystectomy (n = 138)	Non-interval cholecystectomy (n = 67)	*P*-value
Age (years)	64.33 ± 14.61	73.40 ± 12.97	< 0.001
Sex			0.754
Female	65 (47.10%)	30 (44.78%)	
Male	73 (52.90%)	37 (55.22%)	
TG18 grade			< 0.001
Grade I	95 (68.84%)	38 (56.72%)	
Grade II	32 (23.19%)	10 (14.93%)	
Grade III	11 (7.97%)	19 (28.36%)	
Laboratory test			
White blood cell (× 10^9^/L)	12.20 (8.12–15.68)	10.16 (7.32–14.90)	0.956
Direct bilirubin (μmol/L)	11.60 (6.10–19.50)	10.00 (5.47–46.92)	0.099
Total bilirubin (μmol/L)	22.40 (13.60–37.90)	21.80 (11.50–61.70)	0.238
Aspartate aminotransferase (U/L)	26.00 (18.00–53.00)	25.00 (17.00–53.00)	0.566
Alanine aminotransferase (U/L)	26.00 (16.00–60.00)	23.00 (15.00–61.00)	0.164
Serum creatinine (μmol/L)	69.20 (55.70–87.25)	69.30 (51.80–106.90)	0.494
INR	1.22 ± 0.13	1.35 ± 0.80	0.096
Platelet (× 10^9^/L)	189.37 ± 68.98	183.49 ± 76.10	0.587
Albumin stratification (g/L)			0.004
> 35	42 (41.58%)	11 (24.44%)	
28–35	50 (49.50%)	21 (46.67%)	
< 28	9 (8.91%)	13 (28.89%)	
Image performance			
Gallbladder distention (> 4 cm)	129 (93.48%)	65 (97.01%)	0.292
Thickened gallbladder wall (> 3 mm)	135 (97.83%)	64 (95.52%)	0.359
Gallstones	118 (85.51%)	41 (63.08%)	< 0.001
Common bile duct stones	27 (19.57%)	14 (21.54%)	0.744
Common bile duct dilatation (> 6 mm)	34 (24.64%)	17 (25.37%)	0.909
Pericholecystic effusion	70 (50.72%)	33 (49.25%)	0.843
Comorbidity			
Hypertension	48 (34.78%)	30 (44.78%)	0.167
Diabetes	23 (16.67%)	14 (20.90%)	0.460
Cardiovascular and cerebrovascular diseases	40 (28.99%)	34 (50.75%)	0.002
History of malignancy	11 (7.97%)	18 (26.87%)	< 0.001
Liver cirrhosis	2 (1.45%)	0 (0.00%)	0.322
Kidney disease	4 (2.90%)	4 (5.97%)	0.287
Postoperative			
Septic shock	4 (2.90%)	4 (5.97%)	0.287
Transfer to ICU	4 (2.90%)	2 (2.99%)	0.972
Hospitalization days	7 (5–11)	6 (4–10)	0.713
PC-related complications	4 (2.90%)	5 (7.46%)	0.353
Clogged tube	2 (1.45%)	1 (1.49%)	0.981
Dislodged tube	2 (1.45%)	4 (5.97%)	0.072
Recurrence after PC	19 (13.77%)	15 (22.39%)	0.120
All-cause deaths during follow-up	1 (0.72%)	19 (28.36%)	< 0.001

### Analysis of factors influencing patients’ failure to undergo interval cholecystectomy

**Fig. 3:**
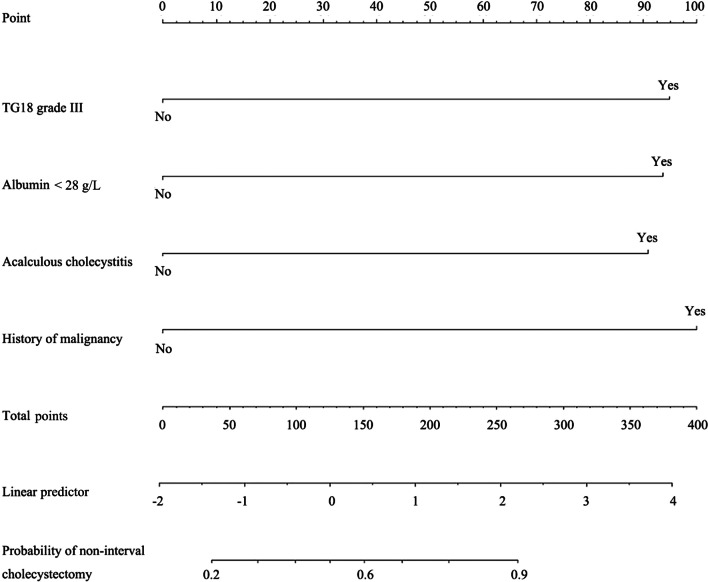
Nomogram for predicting the probability of non-interval cholecystectomy. Top: the predictor points are found on the uppermost point scale that corresponds to each variable. Bottom: the points for all variables are added and translated into the probability of non-interval cholecystectomy

The univariate regression analysis showed that age and having a TG18 grade III status, acalculous cholecystitis, an albumin level < 28 g/L, CCVD, and a history of malignancy were the factors influencing a patient’s failure to undergo interval cholecystectomy. Of these factors, having a TG18 grade III status, acalculous cholecystitis, an albumin level < 28 g/L, and a history of malignancy were independent risk factors for failure to undergo interval cholecystectomy, as per the multiple regression analysis (further details are shown in Table 2). A regression coefficient-based nomogram was constructed from the significant variables (Fig. 3). The presence of a history of malignancy had the highest influence in the nomogram in terms of predicting non-interval cholecystectomy.

**Table 2:** Risk factor analysis for a patient’s failure to undergo interval cholecystectomy

Variables	Statistics	Univariate analysis		Multivariate regression analysis	
		OR (95%CI)	*P *value	OR (95%CI)	*P* value
Age (years)	67.29 ± 14.70	1.05 (1.03, 1.08)	< 0.001	1.02 (0.99, 1.06)	0.202
TG18 grade III	30 (14.63%)	4.57 (2.03, 10.31)	< 0.001	3.83 (1.27, 11.49)	0.017
Acalculous cholecystitis	44 (21.46%)	3.33 (1.67, 6.67)	< 0.001	4.55 (1.59, 12.50)	0.005
Albumin (g/L) < 28	22 (15.07%)	4.15 (1.62, 10.63)	0.003	4.15 (1.09, 15.81)	0.037
Cardiovascular and cerebrovascular diseases	74 (36.10%)	2.52 (1.38, 4.62)	0.003	2.12 (0.87, 5.17)	0.099
History of malignancy	29 (14.15%)	4.24 (1.87, 9.62)	< 0.001	4.65 (1.62, 13.37)	0.004

*CI* confidence interval, *OR* odds ratio, *TG18* Tokyo Guidelines 2018

## Discussion

PC as a bridge to cholecystectomy or as a definitive treatment has been increasingly used as an alternative initial treatment option for patients with AC. However, 60–70% of patients with AC do not undergo interval cholecystectomy after PC [15–18]. In our study, 67 (32.7%) patients did not undergo interval cholecystectomy after PC, which is considerably lower than that in previous studies [15–18].

Interestingly, our study found that in addition to age, a TG18 grade III status, hypoproteinemia, CCVD, and a history of malignancy contributed to the patients’ failure to undergo subsequent cholecystectomy. Among these factors, a TG18 grade III status, hypoproteinemia, and a history of malignancy were independent risk factors that negatively influenced the decision to pursue cholecystectomy. In addition, univariate and multivariate analyses showed that patients with calculous cholecystitis were more likely to undergo cholecystectomy. Although age and the presence of CCVD were not independent risk factors in this study, advanced age and CCVD limit the patients’ ability to undergo surgical procedures that require general anesthesia.

With a Grade III AC, the patient’s overall status usually deteriorates significantly, with one or more organ dysfunctions. Such patients usually have numerous underlying diseases. Even if their cholecystitis is eventually controlled through PC, the patient’s poor physical condition affects their decision to undergo interval cholecystectomy. For patients with a Charlson Comorbidity Index score ≥ 4 or an American Society of Anesthesiologists physical status ≥ 3, the TG18 recommends that conservative treatments should be considered as an alternative to definitive surgery [19].

Gallstones are extremely common [10], and approximately 78.6% of the patients in our study presented with acute calculous cholecystitis. PC can drain the infected bile or pus from the gallbladder and rapidly de-escalate the inflammation and infection status of a patient with cholecystitis. However, PC cannot resolve the high-risk factors for the recurrence of biliary events, such as the presence of gallstones [20, 21]. Although cholecystectomy is the gold standard for the treatment of gallstones and cholecystitis, patients who have undergone PC could still be poor candidates for cholecystectomy and may require permanent gallbladder drainage. PC tube removal after the resolution of AC is an option; however, cholecystitis recurrence rates range from 22 to 41%, mainly due to gallstones [21–23]. Previous attempts to develop minimally invasive definitive treatment options for gallstones have focused on percutaneous gallstone removal, intraluminal sclerosis, and cryoablation [24–26]. These treatment modalities are promising future alternatives to cholecystectomy in patients with permanent indwelling PC tubes.

Hypoproteinemia is an indicator of poor liver function, which is also a factor influencing the patient’s inability to undergo cholecystectomy. This condition also means that the patients are malnourished, which poses a challenge during cholecystectomy performed under general anesthesia.

The expected survival time has a crucial impact on the decision to perform surgery. For patients with a history of malignancy, especially with advanced-stage tumors, a limited survival time and gradual deterioration of their physical condition contribute to their unsuitability to cholecystectomy. In our study, the mortality of the patients who did not undergo cholecystectomy during the follow-up period was as high as 28.36%, which was significantly higher than the mortality in the cholecystectomy group. Malignant tumors were the main cause of death in these patients.

In our study, 21 patients had long-term indwelling PC tubes, whereas 46 patients had their drainage tubes removed. Instead of analyzing these two groups of patients separately, we combined them into the non-interval cholecystectomy group. Furthermore, because of the limitations of retrospective research, for some patients, we could not determine whether the patients had an objective inability to undergo cholecystectomy or whether they had a subjective unwillingness to undergo the procedure. These are the main deficiencies of our research and may have impacted our conclusions. Nevertheless, this is the first study based on a real-world analysis of the factors influencing patients’ failure to undergo cholecystectomy. In addition, we presented a nomogram for individualized risk estimation that allows for the calculation of the numerical probability of undergoing cholecystectomy after PC among patients with AC. We also believe that it is important for clinicians to communicate with patients before PC regarding the possibility of undergoing subsequent cholecystectomy.

## Conclusion

In conclusion, a TG18 grade III status, acalculous cholecystitis, severe hypoproteinemia, and a history of malignancy influence the failure to undergo cholecystectomy after PC among patients with AC.

## Data Availability

The datasets analyzed during the current study are available from the corresponding author on reasonable request.

## Abbreviations

AC: Acute cholecystitis
CCVD: Cardiovascular and cerebrovascular diseases
CI: Confidence interval
LC: Laparoscopic cholecystectomy
OR: Odds ratio
PC: Percutaneous cholecystostomy
TG18: Tokyo Guidelines 2018
